# Supplementary material for: Heat adaptation of phage T7 under an extended genetic code
Source: Virus Evol. 2021 Dec 1;7(2):veab100. doi: 10.1093/ve/veab100 (PMC8923235; doi:10.1093/ve/veab100)
Supplement: veab100_Supp [file veab100_supp.zip › Sequencing_Analysis.zip!/Sequencing_Analysis/S3/evidence/SNP_4.html]

BRESEQ :: Evidence


| Predicted mutation | | | | | | |
| --- | --- | --- | --- | --- | --- | --- |
| evidence | seq id | position | mutation | annotation | gene | description |
| RA | NC\_001604.1 | 18,406 | G→A | intergenic (–/–) | – / – | –/– |

| Read alignment evidence... | | | | | | | | | | | |
| --- | --- | --- | --- | --- | --- | --- | --- | --- | --- | --- | --- |
|  | seq id | position | | ref | new | freq | score (cons/poly) | reads | annotation | genes | product |
| \* | NC\_001604.1 | 18,406 | 0 | G | A | 99.6% | 5521.6 / NA | 1963 | intergenic (–/–) | *–/–* | –/– |
| Reads supporting (aligned to +/- strand):  **ref** base G (4/3);  **new** base A (932/1020);  **total** (938/1025) | | | | | | | | | | | |
| Fisher's exact test for biased strand distribution *p*-value = 7.16e-01 | | | | | | | | | | | |
| Kolmogorov-Smirnov test that lower quality scores support variant *p*-value = 1.00e+00 | | | | | | | | | | | |

Only 100 of 1964 total aligned reads displayed.  

`ATGGTTACTCAGGGATTGCTGGATGGGGTGATACCGCCGAGGACTTCTTGAATAACCCGTTCATAACCGAGCCTAAAACGTCTGTGCTTAAGTCCGGTAAGAACAAAGGCCAAGAGGTTACTAAATGGGTTAAACGCGACCCTGAGCCTCATGAGACGCTTTGGGACTGCATTAAGTCCATTGGCGCGAAGGCTGGTATGACCGAAGAGGATATTATCAAGCAGGGCCAAATGGCTCGAATCCTACGGTTCAACGAGTACAACTTTATTGACAAG‑‑GAGATTTACCTGTGGAGACCGTAGCGTATATTGGTCTGGGTCTTTGTGTTCTCGGAGTGTGCCTCATTTCGTGGGGCCTTT‑GGGACTTAGCCAGAATAATCAAGTCGTTACACGACACTAAGTGATAAACTCAAGGTCCCTAAATTAATACGACTCACTATAGGGAGATAGGGGCCTTTACGATTATTACTTTAAGATTTAACTCTAAGAGGAATCTTTATTATGTTAACACCTATTAACCAATTACTTAAGAACCCTAACGATATTCCAGATGTACCTCGTGCAACCGCTGAGTATCTACAGGTTCGATTCAACTATGCG  >  NC_001604.1/18108‑18703`  

                                                                                                                                                                                                                                                                                                            `|`                                                                                                                                                                                                                                                                                                            
 `tGGTTACTCAGGGATTGCTGGATGGGGTGATACCGCCGAGGACTTCTTGAATAACCCGTTCATAACCGGGCCTAAAACGTCTGTGCTTAAGTCCGGTAAGAACAAAGGACAAGAGGTTACTAAATGGGTTAAACGCGACCCTGAGCCTCATGAGACGCTTTGGGACTGCATTAATTCCATTTGCTCGAAGGCTGGTATGACCGAAGAGGATATTATCAAGCAGGGTCAAATTTCTCTAATCCTATGTTTCAACTAGTACAACTTTATTGACAAG‑‑GAGATTTACCTGTGGAGCCCGGAACGt                                                                                                                                                                                                                                                                                                         >  1:158938/1‑301 (MQ=255)`  
     `taacaaGGGATTGCTGGATGGGGTGATACCGCCGAGGACTTCTTGAATAACCCGTTCATAACCGAGCCTAAAACGTCTGTGCTTAAGTCCGGTAAGAACAAAGGCCAAGAGGTTACTAAATGGGTTAAACGCGACCCTGAGCCTCATGAGACGCTTTGGGACTGCATTAAGTCCATTGGCGCGAAGGCTGGTATGACCGAAGAGGATATTATCAAGCAGGGCCAAATGGCTCGAATCCTACGGTTCAACGAGTACAACTTTATTGACAAG‑‑GAGATTTACCTGTGGAGACCGTAACGTatat                                                                                                                                                                                                                                                                                                     <  1:212253/296‑1 (MQ=255)`  
        `tCAGGGATTGCTGGATGGGGTGATACCGCCGAGGACTTCTTGAATAACCCGTTCATAACCGAGCCTAAAACGTCTGTGCTTAAGTCCGGTAAGAACAAAGGCCAAGAGGTTACTAAATGGGTTAAACGCGACCCTGAGCCTCATGAGACGCTTTGGGACTGCATTAAGTCCATTGGCGCGAAGGCTGGTATGACCGAAGAGGATATTATCAAGCAGGGCCAAATGGCTCGAATCCTACGGTTCAACGAGTACAACTTTATTGACAAG‑‑GAGATTTACCTGTGGAGACCGTAACGTATATTgg                                                                                                                                                                                                                                                                                                  <  1:256528/301‑1 (MQ=255)`  
        `ccAGGGATTGCTGGATGGGGTGATACCGCCGAGGACTTCTTGAATAACCCGTTCATAACCGAGCCTAAAACGTCTGTGCTTAAGTCCGGTAAGAACAAAGGCCAAGAGGTTACTAAATGGGTTAAACGCGACCCTGAGCCTCATGAGACGCTTTGGGACTGCATTAAGTCCATTGGCGCGAAGGCTGGTATGACCGAAGAGGATATTATCAAGCAGGGCCAAATGGCTCGAATCCTACGGTTCAACGAGTACAACTTTATTGACAAG‑‑GAGATTTACCTGTGGAGACCGTAACGTATATTgg                                                                                                                                                                                                                                                                                                  <  1:50966/300‑1 (MQ=255)`  
          `gtttataagagacaggggTGATACCGCCGAGGACGTCTTGAATAACCCGTTCATAACCGAGCCTAAAACGTCTGGGCTTAAGTCCGGTAAGAACAAAGGCCAAGAGGTTACTAAATGGGTTAAACGCGACCCTGGGCCTCATGAGACGCATTGGGACTGCATTAAGTCCATTGGCGCGAAGGCTGGTATGACCGAAGAGGATATTATCAAGCAGGGCCAAATGGCTCGAATCCTACGGTTCAACGAGTACAACTTTATTGACAAG‑‑GAGATTTACCTGTGGAGACCGTAACGTATATTGGTc                                                                                                                                                                                                                                                                                                <  1:195719/287‑1 (MQ=255)`  
          `ggggATTGCTGGATGGGGTGATACCGCCGAGTACTTCTTGAATAACCCGTTCATACCCGAGCCTAAAACGTCTGTGCTTAAGTCCGGTAAGAACAAAGGCCAAGAGATTACTAAATGGGTTAAACGCGACCCTGAGCCTCATGAGACGCGTTGGGACTGCATTAAGTCCATTGGCTCGAAGGCTGGTATGACCGAAGAGGATATTATCAAGCAGGGCCAAATGGCTCGAATCCTACGGTTCAACGAGTACAACTTTATTGACAAG‑‑AAGATTTACCTCTGGAGACCGTAACGTATATTGGTc                                                                                                                                                                                                                                                                                                <  1:314940/300‑1 (MQ=255)`  
               `ttGCTGGATGGGGTGATACCGCCGAGGACTTCTTGAATAACCCGTTCATAACCGAGCCTAAAACGTCTGTGCTTAAGTCCGGTAAGAACAAAGGCCAAGAGGTTACTAAATGGGTTAAACGCGACCCTGAGCCTCATGAGACGCTTTGGGACTGCATTAAGTCCATTGGCGCGAAGGCTGGTATGACCGAAGAGGATATTATCAAGCAGGGCCAAATGGCTCGAATCCTACGGTTCAACGAGTACAACTTTATTGACAAG‑‑GAGATTTACCTGTGGAGACCGTAACGTATATTGGTCTGGGt                                                                                                                                                                                                                                                                                           >  1:299001/1‑301 (MQ=255)`  
                `acagggATGGGGTGAGACCGCCGAGGACTTCTTGAATAACCCGTTCATAACCGAGCCTAAAACGTCTGTGCTTAAGTCCGGTAAGAACAAAGGCCAAGAGGTTACTAAATGGGTTAAACGCGACCCTGAGCCTCATGAGACGCTTTGGGACTGCATTAAGTCCATTGGCGCGAAGGCTGGTATGACCGAAGAGGATATTATCAAGCAGGGCCAAATGGCTCGAATCCTACGGTTCAACGAGTACAACTTTATTGACAAG‑‑GAGATTTACCTGTGGAGACCGTATCGTATATTGGTCTGGGTc                                                                                                                                                                                                                                                                                          <  1:222422/297‑1 (MQ=255)`  
                    `ggATGGGGTGATACCGCCGAGGACTTCTTGAATAACCCGTTCATAACCGAGCCTAAAACGTCTGTGCTTAAGTCCGGTAAGAACAAAGGCCAAGAGGTTACTAAATGGGTTAAACGCGACCCTGAGCCTCATGAGACGCTTTTGGACTGCATTAAGTCCATTGGCGCGAAGGCTGGTATGACCGAAGAGGATATTATCAAGCAGGGCCAAATGGCTCGAATCCTACGGTTCAACGAGTACAACTTTATTGACAAG‑‑GAGATTTACCTGTGGAGACCGTAACGTATAATGGTCTGGGTCtttt                                                                                                                                                                                                                                                                                      >  1:321763/1‑300 (MQ=255)`  
                      `aTGGGGTGATACCACCGAGGACTTCTTGAATAACCCGTTCATAACCGAGCCTAAAACGTCTGTGCTTAAGTCCGGTAAGAACAAAGGCCAAGAGGTTACTAAATGGGTTAAACGCGACCCTGAGCCTCATGAGACGCTTTGGGACTGCATTAAGTCCATTGGCGCGAAGGCTGGTATGACCGAAGAGGATATTATCAAGCAGGGCCAAATGGCTCGAATCCTACGGTTCAACGAGTACAACTTTATTGACAAG‑‑GAGATTTACCTGTGGAGACCGTAACGTATATTGGTCTGGGTCTTtgtg                                                                                                                                                                                                                                                                                    <  1:174978/301‑1 (MQ=255)`  
                         `gggTGATACCGCCGAGGACTTCTTGAATAACCCGTTCATAACCGAGCCTAAAACGTCTGTGCTTAAGTCCGGTAAGAACAAAGGCCAAGAGGTTACTAAATGGGTTAAACGCGACCCTGAGCCTCATGAGACGCTTTGGGACTGCATTAAGTCCATTGGCGCGAAGGCTGGTATGACCGAAGAGGATATTATCAAGCAGGGCCAAATGGCTCGAATCCTACGGTTCAACGAGTACAACTTTATTGACAAG‑‑GAGATTTACCTGTGGAGACCGTAACGTATATTgctgtctcttatacacatc                                                                                                                                                                                                                                                                                 >  1:197896/1‑283 (MQ=255)`  
                         `gggTGATACCGCCGAGGACTTCTTGAATAACCCGTTCATAACCGAGCCTAAAACGTCTGTGCTTAAGTCCGGTAAGAACAAAGGCCAAGAGGTTACTAAATGGGTTAAACGCGACCCTGAGCCTCATGAGACGCTTTGGGACTGCATTAAGTCCATTGGCGCGAAGGCTGGTATGACCGAAGAGGATATTATCAAGCAGGGCCAAATGGCTCGAATCCTACGGTTCAACGAGTACAACTTTATTGACAAG‑‑GAGATTTACCTGTGGAGACCGTAACGTATATTGGTcctgtctcttatacac                                                                                                                                                                                                                                                                                 >  1:292308/1‑286 (MQ=255)`  
                              `aTACCGCCGAGGACTTCTTGAATAACCCGTTCATAACCGAGCCTAAAACGTCTGTGCTTAAGTCCGGTAAGAACAAAGGCCAAGAGGTTACTAAATGGGTTAAACGCGACCCTGAGCCTCATGAGACGCTTTGGGACTGCATTAAGTCCATTGGCGCGAAGGCTGGTATGACCGAAGAGGATATTATCAAGCAGGGCCAAATGGCTCGAATCCTACGGTTCAACGAGTACAACTTTATTGACAAG‑‑GAGATTTACCTGTGGAGACCGTAACGTATATTGGTCTGGGTCTTTGTGTTCTCggt                                                                                                                                                                                                                                                                            >  1:218981/1‑300 (MQ=255)`  
                                 `ccgccgAGGACTTCTTGAATAACCCGTTCATAACCGAGCCTAAAACGTCTGTGCTTAAGTCCGGTAAGAACAAAGGCCAAGAGGTTACTAAATGGGTTAAACGCGACCCTGAGCCTCATGAGACGCTTTGGGACTGCATTAAGTCCATTGGCGCGAAGGCTGGTATGACCGAAGAGGATATTATCAAGCAGGGCCAAATGGCTCGAATCCTACGGTTCAACGAGTACAACTTTATTGACAAG‑‑GAGATTTACCTGTGGAGACCGTAACGTATATTGGTCTGGGTcctgtctcttatacccat                                                                                                                                                                                                                                                                         >  1:131232/1‑284 (MQ=255)`  
                                   `gccgAGGACTTCTTGAATAACCCGTTCATAACCGAGCCTAAAACGTCTGTGCTTAAGTCCGGTAAGAACAAAGGCCAAGAGGTTACTAAATGGGTTAAACGCGACCCTGAGCCTCATGAGACGCTTTGGGACTGCATTAAGTCCATTGGCGCGAAGGCTGGTATGACCGAAGAGGATATTATCAAGCAGGGCCAAATGGCTCGAATCCTACGGTTCAACGAGTACAACTTTATTGACAAG‑‑GAGATTTACCTGTGGAGACCGTAACGTATATTGGTCTGGGTCTTTGTGTTCTCGGAgctgt                                                                                                                                                                                                                                                                       >  1:267402/1‑297 (MQ=255)`  
                                            `ttcttGAATAACCCGTTCATAACCGAGCCTAAAACGTCTGTGCTTAAGTCCGGTAAGAACAAAGGCCAAGAGGTTACTAAATGGGTTAAACGCGACCCTGAGCCTCATGAGACGCTTTGGGACTGCATTAAGTCCATTGGCGCGAAGGGTGGTATGACCGAAGAGGATATTATCAAGCAGGGCCAAATGGCTCGAATCCTACGGTTCAACGAGTACAACTTTATTGACAAG‑‑GAGATTTACCTGTGGAGACCGTAACGTATATTGGTCTGGGTCTTTGTGTTCTCGGAGTGTGCCTCATTTc                                                                                                                                                                                                                                                              <  1:61327/301‑1 (MQ=255)`  
                                                `tGAATAACCCGTTCATAACCGAGCCTAAAACGTCTGTGCTTAAGTCCGGTAAGAACAAAGGCCAAGAGGTTACTAAATGGGTTAAACGCGACCCTGAGCCTCATGAGACGCTTTGGGACTGCATTAAGTCCATTGGCGCGAAGGCTGGTATGACCGAAGAGGATATTATCAAGCAGGGCCAAATGGCTCGAATCCTACGGTTCAACGAGTACAACTTTATTGACAAG‑‑GAGATTTACCTTTGGAGACCGTAAAGTATATTGGTCTGGGTCTTTGTGTTCTCGGAGTGTGCCTACTTTCGTgg                                                                                                                                                                                                                                                          >  1:93763/1‑301 (MQ=255)`  
                                                       `cccGTTCATAACCGAGCCTAAAACGTCTGTGCTTAAGTCCGGTAAGAACAAAGGCCAAGAGGTTACTAAATGGGTTAAACGCGACCCTGAGCCTCATGAGACGCTTTGGGACTGCATTAAGTCCATTGGCGCGAAGGCTGGTATGACCGAAGAGGATATTATCAAGCAGGGCCAAATGGCTCGAATCCTACGGTTCAACGAGTACAACTTTATTGACAAG‑‑GAGATTTACCTGTGGAGACCGTAACGTATATTGGTCTGGGTCTTTGTGTTCTCGGAGTGTGccctgtctcttatacacatc                                                                                                                                                                                                                                                   >  1:214275/1‑283 (MQ=255)`  
                                                          `gTTCATAACCGAGCCTAAAACGTCTGTGCTTAAGTCCGGTAAGAACAAAGGCCAAGAGGTTACTAAATGGGTTAAACGCGACCCTGAGCCTCATGAGACGCTTTTGGACTGCATTAAGTCCATTGGTGCGAAGGCTGGTATGACCGAAGAGGATATTATCAAGCAGGGCCAAATGGCTCGAATCCTACGGTTCAACGAGTACAACTTTATTGACAAG‑‑GAGATTTACCTGTGGAGACCGTAACGTATATTGGTCTGGGTCTTTGTGTTCTCGGAGTGTTCCTcctgtctcttatacacatct                                                                                                                                                                                                                                                >  1:177196/1‑282 (MQ=255)`  
                                                          `gTTCATAACCGAGCCTAAAACGTCTGTGCTTAAGTCCGGTAAGAACAAAGGCCAAGAGGTTACTAAATGGGTTAAACGCGACCCTGAGCCTCATGAGACGCTTTGGGACTGCATTAAGTCCATTGGCGCGAAGGCTGGTATGACCGAAGAGGATATTATCAAGCAGGGCCAAATGGCTCGAATCCTACGGTTCAACGAGTACAACTTTATTGACACG‑‑GAGATTTACCTGTGGAGACCGTAACGTATATTGGTCTGGGTCTTTGTGTTCTCGGAGTGTGCCTCATTTCGTTGGGCCTTT‑ggg                                                                                                                                                                                                                                               >  1:128324/1‑301 (MQ=255)`  
                                                            `tCATAGCCGAGCCTAAAACGTCTGTGCTTAAGTCCGGTAAGAACAAAGGCCAAGAGGTTACTAAATGGGTTAAACGCGACCCTGAGCCTCATGAGACGCTATGGGACTGCATTAAGTCCATTGGCGCGAAGGCTGGTATGACCGAAGAGGATATTATCAAGCAGGGCCAAATGGCTCGAATCCTACGGTTCAACGAGTACAACTTTATTGACAAG‑‑GAGATTTACCTGTGGAGACCGTAACGTATATTGGTCTGGGTCTTTGTGTTCTCGGAGTGTGCCTCATTTCGTGGGGCCTTT‑GGGAc                                                                                                                                                                                                                                             <  1:143036/301‑1 (MQ=255)`  
                                                               `tAACCGAGCCTAAAACGTCTGTGCTTAAGTCCGGTAAGAACAAAGGCCAAGAGGTTACTAAATGGGTTAAACGCGACCCTGAGCCTCATGAGACGCTTTGGGACTGCATTAAGTCCATTGGCGCGAAGGCTGGTATGACCGAAGAGGATATTATCAAGCAGGGCCAAATGGCTCGAATCCTACGGTTCAACGAGTACAACTTTATTGACAAG‑‑GAGATTTACCTGTGGAGACCGTAACGTCTATTGGTCTTGGTCTTTGTTTTCTCGGAGTGTGCCTCATTTCGTTGGGCCTTT‑GGGACttt                                                                                                                                                                                                                                          >  1:166333/1‑300 (MQ=255)`  
                                                                         `tAAAACGTCTGTTCTTAAGTCCGGTAAGAACAAAGGCCAAGAGGTTACTAAATGGGTTAAACGCGACCCTGAGCCTCATGAGACGCTTTGGGACTGCATTAAGTCAATTGGCGCGAAGGCTGGTATGACCGAAGAGGATATTATCAAGCAGGGCCACATGGCTCGAATCCTACGTTTCAACTAGTACAACTTTATTGACAAG‑‑GAGATTTCACTATAGAGACCGTAACGTATATTGGTCTGGGTCTTTGTGGTCTCGGAGTGTTCCTCCTTTCGTTGTGCCttgttgtcttattcagtataa                                                                                                                                                                                                                                 >  1:209361/1‑282 (MQ=255)`  
                                                                            `aaCGTATGTGCTTAAGTCCGGTAAGAACAAAGGCCAAGAGGTTACTAAATGGGTTAAACGCGACCCTGAGCCCCATGAGACGCTTTGGGACTGCATTAAGTCCATTGGCGCGAAGGCTGGTATGACCGAAGAGGATATTATCAAGCAGGGCCAAATGGCTCGAATCCTACGGTTCAACGAGTACAACTTTATTGACAAG‑‑GAGATTTACCTGTGGAGACCGTAACGTATATTGGTCTGGGTCTTTGTGTTCTCGGAGTGTGCCTCATTTCGTGGGGCCTTT‑GGGACTTAGCCAGAATAATCa                                                                                                                                                                                                                             <  1:310827/301‑1 (MQ=255)`  
                                                                                   `gtgCTTAAGTCCGGTAAGAACAAAGGCCAAGAGGTTACTAAATGGGTTAAACGCGACCCTGAGCCTCATGAGACGCTTTGGGACTGCATTAAGTCCATTAGCGCGAAGGCTGGTATGACCGAAGAGGATATTATCAAGCAGGGCCAAATGGCTCGAATCCTACGGTTCAACGAGTACAACTTTATTGACAAG‑‑GAGATTTACCTGTGGAGACCGTAACGTATATTGGTCTGGGTCTTTGTGTTCTCGGAGTGTGCCTCATTTCGTGGGGCCTTT‑GGGACTTAGCCCGAATATTCAAGTCggt                                                                                                                                                                                                                      >  1:279452/1‑299 (MQ=255)`  
                                                                                   `gtacTTAAGTCCGGTAAGAACAAAGGCCAAGAGGTTACTAAATGGGTTAAACGCGACCCTGAGCCTCATGAGACGCTTTGGGACTGCATTAAGTCCATTGGCGCGAAGGCTGGTATGACCGAAGAGGATATTATCAAGCAGGGCCAAATGGCTCGAATCCTACGGTTCAACGAGTACAACTTTATTGACAAG‑‑GAGATTTACCTGTGGAGACCGTAACGTATATTGGTCTGGGTCTTTGTGTTCTCGGAGTGTGCCTCATTTCGTGGGGCCTTT‑GGGACTTAGCCAGAATAATCAAGTCGtt                                                                                                                                                                                                                      >  1:172718/4‑301 (MQ=255)`  
                                                                                      `cTTAAGTCCGGTAAGAACAAAGGCCAAGAGGTTACTAAATGGGTTAAACGCGACCCTGAGCCTCATGAGACGCTTTGGGACTGCATTAAGTCCATTGGCGCGAAGGCTGGTATGACCGAAGAGGATACTATCAAGCAGGGCCAAATGGCTCGAATCCTACGGTTCAACGAGTACAACTTTATTGACAAG‑‑GAGATTTACCTGTGGAGACCGTAACGTCTATTGGTCTGGGTCTTTGTGTTCTCGGAGTGTGCCTCATTTCGTGGGGCCTTT‑GGGTCTTAGCCAGAATAATCACGTCGTTaca                                                                                                                                                                                                                   >  1:186274/1‑301 (MQ=255)`  
                                                                                       `ttAAGTCCGGTAAGAACAAAGGCCAAGAGGTTACTAAATGGGTTAAACGCGACCCTGAGCCTCATGAGACGCTTTGGGACTGCATTAAGTCCATTGGCGCGAAGGCTGGTATGACCGAAGAGGATATTATCAAGCAGGGCCAAATGGCTCGAATCCTACGGTTCAACGAGTACAACTTTATTGACAAG‑‑GAGATTTACCTGTGGAGACCGTAACGTATATTGGTCTGGGTCTTTGTGTTCTCGGAGTGTGCCTCATTTCGTGGGGCCTTT‑GGGACTTAGCCAGAATAATCAAGTCGTTacac                                                                                                                                                                                                                  <  1:288661/301‑1 (MQ=255)`  
                                                                                       `tgaaGTCCGGTAAGAACAAAGGCCAAGAGGTTAATAAATGGGTTAAACGCGACCCTGAGCATCATGAGACGCTTTGGGACTGCATTAAGTACATTGGCGCGAAGGCTGGTAGGACCGAAGAGGATATTATCAAGCAGGGCCAAATGGCTCGAATCCTACGGTTCAACGAGTACAACTTTATTGACAAG‑‑GAGATTTAACTGTGGAGACCGGAACGTATATTGGTCTGGGTCTTTGTGTTCTCGGAGTGTGCCACATTTCGTGGGGCCTTT‑GGGACTTAGCCAGAATAATCAAGTCGTTacac                                                                                                                                                                                                                  <  1:48209/299‑1 (MQ=255)`  
                                                                                        `tAAGTCCGGTAAGAACAAAGGCCAAGAGGTTACTAAATGGATTAAACGCGACCCTGAGCCTCATGAGACGCTTTGGGACTGCATTAAGTCCATTGGCGCGAAGGCTGGTATGACCGAAGAGGATATTATCAAGCAGGGCCAAATGGCTCGAATCCTACGGTTCAACGAGTACAACTTTATTGACAAG‑‑GAGATTTACCTGTGGAGACCGTAACGTATATTGGTCTGGGTCTTTGTGTTCTCGGAGTGTGCCTCATTTCGTGGGGCCTTT‑GGGACTTAGCCAGAATAATCAAGTCGTTACACg                                                                                                                                                                                                                 <  1:168569/301‑1 (MQ=255)`  
                                                                                           `gTCCGGTAAGAACAAAGGCCAAGAGGTTACTAAATGGGTTAAACGCGACCCTGAGCCTCATGAGACGCTTTGGGACTGCATTAAGTCCATTGGCGCGAAGGCTGGTATGACCGAAGAGGATATTATCAAGCAGGGCCAAATGGCTCGAATCCTACGGTTCAACGAGTACAACTTTATTGACAAG‑‑GAGATTTACCTGTGGAGACCGTAACGTATATTGGTCTGGGTCTTTGTGTTCTCGTAGTGTGCCTCATTTCGTGGGGCCTTT‑GGGACTTAGCCAGAATAATCAAGTCGTTACACGaca                                                                                                                                                                                                              >  1:246234/1‑301 (MQ=255)`  
                                                                                             `ccGGTAAGAACAAAGGCCAAGAGGTTACTAAATGGGTTAAACGCGACCCTGAGCCTCATGAGACGCTTTGGGACTGCATTAAGTCCATTGGCGCGAAGGCTGGTATGACCGAAGAGGATATTATCAAGCAGGGCCAAATGGCTCGAATCCTACGGTTCAACGAGTACAACTTTATTGACAAG‑‑GAGATTTACCTGTGGAGACCGTAACGTATATTGGTCTGGGTCTTTGTGTTCTCGGAGTGTGCCTCATTTCGTGGGGCCTTT‑GGGACTTAGCCAGAATAATCAAGTCGTTACACGACACt                                                                                                                                                                                                            >  1:325288/1‑301 (MQ=255)`  
                                                                                                        `aaaGGCCAAGAGGTTACTAAATGGGTTAAACGCGACCCTGGGCCTCATGAGAAGCTTTGGGACTGCATTAAGTCCATTGGCGCGAAGGCTGGTATGACCGAAGAGGATATTATCAAGCAGGGCCAAATGGCTCGAATCCTACGGTTCAACGAGTACAACTTTATTGACAAG‑‑GAGATTTACCTGTGGAGACCGTAACGTATATTGGTCTGGGTCTTTGTGTTCTCGGAGTGTGCCTCATTTCGTGGGGCCTTT‑GGGACTTAGCCAGAATAATCAAGTCGTTACACGACACTAAGTGATAAAc                                                                                                                                                                                                 <  1:181645/301‑1 (MQ=255)`  
                                                                                                            `gCCTAGAGGTTACTAAATGGGTTAAACGCGACCCTGAGCCTCATGAGACGCTTTGGGACTGCATTAAGTCCATTGGCGCGAAGGCTGGTATGACCGAAGAGGATATTATCAAGCAGGGCCAAATGGCTCGAATCCTACGGTTCAACGAGTACAACTTTATTGACAAG‑‑GAGATTTACCTGTGGAGACCGTAACGTATATTGGTCTGGGTCTTTGTGTTCTCGGAGTGTGCCTCATTTCGTGGGGCCTTT‑GGGACTTAGCCAGAATAATCAAGTCGTTACACGACACTAAGTGATAAACTCaa                                                                                                                                                                                             >  1:63841/1‑301 (MQ=255)`  
                                                                                                            `gCCAAGAGGTTACTAAATGGGTTAAACGCGACCCTGAGCCTCATGAGACGCTTTGGGACTGCATTAAGTCCATTGGCGTGAAGGCTGGTATGACCGAAGAGGATATTATCAAGCAGGGCCAAATGGCTCGAATCCTACGGTTCAACGAGTACAACTTTATTGACAAG‑‑GAGATTTACCTGTGGAGACCGTAACGTATATTGGTCTGGGTCTTTGTGTTTTCGGAGTGTGCCTCATTTCGTGGGTCCTTT‑GGGACTTAGCCAGAATAATCAAGTCGGTACACCACACTAAGTGTTAAACTCaa                                                                                                                                                                                             >  1:139349/1‑301 (MQ=255)`  
                                                                                                              `cAAGAAGTTACTAAATGGGTTAAACGCGACCCTGAGCCTCATGAGACGCTCTGGGACTGCATTAAGTCCATTGGCGCGAAGGCGGGTATGACCGAAGAGGATATTATCAAGCAGGGCCAAATGGCTCGAATCCTACGGTTCAACGAGTACAACTTTATTGACAAG‑‑GAGATTTACCTGTGGAGACCGTAACGTATATTGGTCTGGGTCTTTGTGTTCTCGGAGTGTGCCTCATTTCGTGGGGCCTTT‑GGGACTTAGCCAGAATAATCAAGTCGTTACACGACACTAAGTGATAAACTCAAgg                                                                                                                                                                                           <  1:31887/301‑1 (MQ=255)`  
                                                                                                                 `gagGTTACTAAATGGGTTAAACGCGACCCTGAGCCTCATGCGACGCTTTGGGACTGCATTAAGTCCATTGGCGCGAAGGCTGGTATGACCGAAGCGGATATTATCAAGCAGGGCCAAATGTATCGAATCCTACGGTTCAAAGAGTACACCTTTATTGACAAG‑‑GAGATTTACCTGTGGAGACAGTAACGTATATTGGTCTGGGTCTTTGTGTTCTCGGAGTGTGCCTCATTTCGTGGGGCCTTT‑TGGACTTAGCCAGAATAATCAAGTCGTTAACCGACACTAGCTGATAAAATCAAGGTcc                                                                                                                                                                                        >  1:286062/1‑301 (MQ=255)`  
                                                                                                                     `ttACTAAATGGGTTAAACGCGACCCTGAGCCTCATGAGACGCTTTGGGACTGCATTAAGTCCATTGGCGCGAAGGCTGGTATGACCGAAGAGGATATTATCAAGCAGGGCCAAATGGCTCGAATCCTACGGTTCAACGAGTACAACTTTATTGACAAG‑‑GAGATTTACCTGTGGAGACCGTAACGTATATTGGTCTGGGTCTTTGTGTTATCGGAGTGTGCCTCATTTCGTGGGGCCTTT‑GGGACTTAGCCAGAATAATCAAGTCGTTACACGACACTAAGTGATAAACTCAAGGTCCCTaa                                                                                                                                                                                    >  1:236641/1‑301 (MQ=255)`  
                                                                                                                        `cTAAATGGGTTAAACGCGACCCTGAGCCTAATGAGACGCTTTGGGACTGCATTAAGTCCATTGGCGCGAAGGCTGGTATGACCGAAGAGGATATTATCAAGCAGGGCCAAATGGCTCGAATCCTACGGTTCAACGAGTACAACTTTATTGACAAG‑‑GAGATTTACCTGTGGAGACCGTAACGTATATTGGTCTGGGTCTTTGTGTTCTCGGAGTGTGCCTCATTTCGTGGGGCCTTT‑GGGACTTAGCCAGAATAATCAAGTCGTTACACGACACTAAGTGATAAACTCAAGGTCCCTAAAtt                                                                                                                                                                                 <  1:307164/301‑1 (MQ=255)`  
                                                                                                                            `ttttataagggaccgcccTGAGCCTCATGAGACGCTTTGGGACTGCATTAAGTCCATTGGCGCGAAGGCTGGTATGACCGAAGAGGATATTATCAAGCAGGGCCAAATGGCTCGAATCCTACGGTTCAACGAGTACAACTTTATTGACAAG‑‑GAGATTTACCTGTGGAGACCGTAACGTATATTGGTCTGGGTCTTTGTGTTCTCGGAGTGTGCCTCATTTCGTGGGGCCTTT‑GGGACTTAGCCAGAATAATCAAGTCGTTACACGACACTAAGTGATAAACTCAAGGTCCCTAAATTAATa                                                                                                                                                                             <  1:37150/286‑1 (MQ=255)`  
                                                                                                                             `ggggTTAAACGCGACCCTGAGCCTCATGAGACGCTTTGGGACTGCATTAAGTCCATTGGCGCGAAGGCTGGTATGACCGAAGAGGATATTATCAAGCAGGGCCAAATGGCTCGAATCCTACGGTTCAACGAGTACAACTTTATTGACAAG‑‑GAGATTTACCTGTGGAGACCGTAACGTATATTGGTCTGGGTCTTTGTGTTCTCGGAGTGTGCCTCATTTCGTGGGGCCTTT‑GGGACTTAGCCAGAATAATCAAGTCGTTACACGACACTAAGTGATAAACTCGAGGTCCCTAAATTAATAc                                                                                                                                                                            <  1:145607/300‑1 (MQ=255)`  
                                                                                                                               `ggTTAAACGCGACCCTGAGCCTCATGAGACGCTTTGGGACTGCATTAAGTCCATTGGCGCGAAGGCTGGTATGACCGAAGAGGATATTATCAAGCAGGGCCAAATGGCTCGAATCCTACGGTTCAACGAGTACAACTTTATTGACAAG‑‑GAGATTTACCTGTGGAGACCGTAACATATATTGGTCTGGGTCTTTGTGTTCTCGGAGTGTGCCTCATTTCGTGGGGCCTTT‑GGGACTTAGCCAGAATAATCAAGTCGTTACACGACACTAAGTGATAAACTCAAGGTCCCTAAATTAATACGa                                                                                                                                                                          >  1:243983/1‑301 (MQ=255)`  
                                                                                                                                          `aCCCTGAGCCTCATGAGACGCTTTGGGACTGCATTAAGTCCATTGGCGCGAAGGCTGGTATGACCGAAGAGGATATTATCAAGCAGGGCCAAATGGCTCGAATCCTACGGTTCAACTAGTACAACTTTATTGACAAG‑‑GAGATTTACCTGTGGAGACCGTAACGTATATTGGTCTGGGTCTTTGTGTTCTCGGAGTGTGCCTCATTTCGTGGGGCCTTT‑GGGACTTAGCCAGAATAATCAAGTCGTTACACGACCCCAAGTGATAAACTCAAGGGCCCTAAATTAATACGACTccccaactt                                                                                                                                                               >  1:136750/1‑293 (MQ=255)`  
                                                                                                                                           `cccTGCTCCTCATGAGACGCTTTGGGACTACATTAAGTCCATTGGCTCGAAGGCTGGTATGACCTAAGAGGATATTATCAAGCAGTGCCAAATGGCTCGAATCCTACGGTTCAACGAGTACAACTTTATTGTCAAG‑‑GATATTTACCTGTGGAGCCCGTAACTTATATTGGTCTGTGTCTTTGTGTTCTCGGAGTTTGCCTCATTTCGTGGGGCCTTT‑GGCACTTAGCCAGAATAATCAAGTCGTTACACGACACTAAGTGATAAACTCAAGGTCCCTAAATTAATACGACTCACTATAggg                                                                                                                                                              <  1:100000/301‑1 (MQ=255)`  
                                                                                                                                           `cccTGAGCCTCATGAGACGCTTTGGGACTGCATTAAGTCCATTGGCGCGAAGGCTGGTATGACCGAAGAGGATATTATCAAGCAGGGCCAAATGGCTCGAATCCTACGGTTCAACGAGTACAACTTTATTGACAAG‑‑GAGATTTACCTGTGGAGACCGTAACGTATATTGGTCTGGGTCTTTGTGTTCTCGGAGTGTGCCTCATTTCGTGGGGCCTTT‑GGGACTTAGCCAGAATAATCAAGTCGTTACACGACACTAAGTGATAAACTCAAGGTCCCTAAATTAATACGACTCACTATAggg                                                                                                                                                              <  1:114296/301‑1 (MQ=255)`  
                                                                                                                                           `cccTGAGCCTCATGAGACGCTTTGGGACTGCATTAAGTCCATTGGCGCGAAGGCTGGTATGACCGAAGAGGATATTATCAAGCAGGGCCAAATGGCTCGAATCCTACGGTTCAACGAGTACAACTTTATTGACAAG‑‑GAGATTTACCTGTGGAGACCGTAACGTATATTGGTCTGGGTCTTTGTGTTCTCGGAGTGTGCCTCATTTCGTGGGGCCTTT‑GGGACTTAGCCAGAATAATCAAGTCGTTACACGACACTAAGTGATAAACTCAAGGTCCCTAAATTAATACGAATAACTATAggg                                                                                                                                                              >  1:46111/1‑301 (MQ=255)`  
                                                                                                                                           `cccTGAGCCTCATGAGACGCTTTGGGACTGAATTAAGTCCATTGGCGCGAAGGCTGGTATGACCGAAGAGGATATTATCAAGCAGGGCCAAATGGCTCGAATCCTACGGTTCAACGAGTACAACTTTATTGACAAG‑‑GAGATTTACCTGTGGAGACCGTAACGTATATTGGTCTGGGTCTTTGTGTTCTCGGAGTGTGCCTCATTTCGTGGGGCCTTT‑GGGACTTAGCCAGAATAATCAAGTCGTTACACGACACTAAGTGATAAACTCAAGGTCCCTAAATTAATACGACTCACTATAggg                                                                                                                                                              <  1:42787/301‑1 (MQ=255)`  
                                                                                                                                           `agatgtgtataagagacagCTTTGGGACTGCATTAAGTCCATTGGCTCGAAGGCTGGTATGACCGAAGAGGATATTATCAAGCAGGGCCAAATGGCTCGAATCCTACGGTTCAACGAGTACAACTTTATTGACAAG‑‑GAGATTTACCTGTGGAGACCGTAACGTATATTGGTCTGGGTCTTTGTGTTCTCGGAGTGTGCCTCATTTCGTGGGGCCTTT‑GGGACTTAGCCAGAATAATCAAGTCGTTACACGACACTAAGTGATAAACTCAAGGTCCCTAAACTAATACGACTCACTATAggg                                                                                                                                                              <  1:118322/283‑1 (MQ=255)`  
                                                                                                                                            `tgtataagagacaggaCGCTTTGGGACTGCATTAAGGCCATTGTCGCGAAGGCTGGGATGACCGAAGAGGATATTATCAAGCAGGGCCAAATGGCTCGAATCCTACGGTTCAACGAGTACAACTTTATTGACAAG‑‑GAGATTTACCTGTGGAGACCGTAACGTATATTGGTCTGGGTCTTTGTGTTCTCGGAGTGTGCCTCATTTCGTGGGGCCTTT‑GGGACTTAGCCAGAATAATCAAGTCGTTACACGACACTAAGTGATAAACTCAAGGTCCCTAAATTAATACGACTCACTATAGGga                                                                                                                                                             <  1:24917/287‑1 (MQ=255)`  
                                                                                                                                             `cTGAGCCTCATGAGACGCTTTGGGACTGCATTAAGTCCATTGGCGCGAAGGCTGGTATGACCGAAGAGGATATTATCAAGCAGGGCCAAATGGCTCGAATCCTACGGTTCAACGAGTACAACTTTATTGACAAG‑‑GAGATTAACCTGTGGAGACCGTAACGTATATTGGTCTGGGTCTTTGTGTTCTCGGAGTGTGCCTCATTTCGTGGGGCCTTT‑GGGACTTAGCCAGAATAATCAAGTCGTTACACGACACTAAGTGATAAACTCAAGGTCCCTAAATTAATACGACTCACTATAGGgag                                                                                                                                                            <  1:229574/301‑1 (MQ=255)`  
                                                                                                                                               `gAGCCTCATGAGACGCTTTGGTACTGCATTAAGTCCATTGGCGCGAAGGCTGGTATGACCGAAGAGGATATTATCAAGCAAGGCCAAATGGCTCGAATCCTACGGTTCAACGAGTACAACTTTATTGACAGG‑‑GAGATTTACCTGTGGAGACCGTAACGTATATTGGTCTGGGTCTTTGTGTTCTCGGAGTGTGCCTCATTTCGTGGGTCCTTT‑TGGACTTAGCCAGAATAATCAAGTCGTTACACGCCACTAAGTGATAAACTCAAGGTCCCTAAATTAATCCGACTCACTATAGGgagct                                                                                                                                                          >  1:152569/1‑299 (MQ=255)`  
                                                                                                                                               `gAGCCTCATGAGACGCTTTGGGACTGCATTAAGTCCATTGGCGCGAAGTCTGGTATGACCGAAGAGGATATTATCAAGCAGGGCCAAATGGCTCGAATCCTACGGTTCAACGAGTACAACTTTATTGACAAG‑‑GAGATTTACCTGTGGAGACCGTAACGTATATTGGTCTGGGTCTCTGTGTTCTCGGAGTGTGCCTCATTTCGTGGGGCCTTT‑GGGACTTAGCCAGAATAATCAAGTCGTTACACGACACTAAGTGATAAACTCAAGGTCCCTAAATTAATACGACTCACTATAGGgagct                                                                                                                                                          >  1:204517/1‑299 (MQ=255)`  
                                                                                                                                               `gAGCCTCATGAGACGCTTTGGGACTGCATTAAGTCCATTGGCGCGAAGGCTGGTATGACCGAAGAGGATATTATCAAGCAGGGCCAAATGGCTCGAATCCTACGGTTCAACGAGTACAACTTTATTGACAAG‑‑GAGATTTACCTGTGGAGACCGTAACGTATATTGGTCTGGGTCTTTGTGTTCTCGGAGTGTGCCTCATTTCGTGGGGCCTTT‑GGGACTTAGCCAGAATAATCAAGTCGTTACACGACACTAAGTGATAAACTCAAGGTCCCTAAATTAATACGACTCACTATAGGGAGAt                                                                                                                                                          >  1:86189/1‑301 (MQ=255)`  
                                                                                                                                                 `aatgtgtataagagacagggACTGCATTAAGTCCATTGGCGCGAAGGCTGGTATGACCGAAGAGGATATTATCAAGCAGGGCCAAATGGCTCGAATCCTACGGTTCAACGAGTACAACTTTATTGACAAGGAGAGATTTACCTGTGGAGACCGTAACGTATATTGGTCTGGGTCTTTGTGTTCTCGGAGTGTGCCTCATTTCGTGGGGCCTTT‑GGGACTTAGCCAGAATAATCAAGTCGTTACACGACACTAAGTGATAAACTCAAGGTCCCTAAATTAATACGACTCACTATAGGGAGAt                                                                                                                                                          <  1:160362/284‑1 (MQ=255)`  
                                                                                                                                                     `cATGAGACGCTTTGGGACTGCATTAAGTCCATTGGCGCGAAGGCTGGTATGACCGAAGAGGATATTATCAAGCAGGGCCAAATGGCTCGAATCCTACGGTTCAACGAGTACAACTTTATTGACAAG‑‑GAGATTTACCTGTGGAGACCGTAACGTATATTGGTCTGGGTCTTTGTGTTCTCGGAGTGTGCCTCATTTCGTGGGGCCTTT‑GGGACTTAGCCAGAATAATCAAGTCGTTACACGACACTAAGTGATAAACTCAAGGTCCCTACATTAATACGACTCACTCTAGGGCGATAGGGGc                                                                                                                                                    >  1:337730/1‑301 (MQ=255)`  
                                                                                                                                                     `cATGAGACGCTTTGGGACTGCATTAAGTCCATTGGCGCGAAGGCTGGTATGACCGAAGAGGATATTATCAAGCAGGGCCAAATGGCTCGAATCCTACGGTTCAACGAGTACAACTTTATTGACAAG‑‑GAGATTTACCTGTGGAGACCGTAACGTATATTGGTCTGGGTCTTTGTGTTCTCGGAGTGTGCCTCATTTCGTGGGGCCTTT‑GGGACTTAGCCAGAATAATCAAGTCGTTACACGACACTAAGTGATAAACTCAAGGTCCCTAAATTAATACGACTCACTATAGGGAGATAGGGGc                                                                                                                                                    <  1:216966/301‑1 (MQ=255)`  
                                                                                                                                                             `gCTTTGGGACTGCATTAAGTCCATTGGCGCGAAGGCTGGTATGACCGAAGAGGATATTATCAAGCAGGGCCAAATGGCTCGAATCCTACGGTTCAACGAGTACAACTTTATTGACAAG‑‑GAGATTTACCTGTGGAGACCGTAACGTATATTGGTCTGGGTCTTTGTGTTCTCGGAGTGTGCCTCATTTCGTGGGGCCTTT‑GGGACTTAGCCAGAATAATCAAGTCGTTACACGACACTAAGTGATAAACTCAAGGTCCCTAAATTAATACGACTCACTATAGGGAGATAGGGGCCTTTACGa                                                                                                                                            <  1:260614/301‑1 (MQ=255)`  
                                                                                                                                                              `cTTTGGGACTGCATTAAGTCCATTGGCGCGAAGGCTGGTATGACCGAAGAGGATATTATCAAGCAGGGCCAAATGGCTCGAATCCTACGGTTCAACGAGTACAACTTTATTGACAAG‑‑GAGATTTACCTGTGGAGACCGTAACGTATATTGGTCTGGGTCTTTGTGTTCTCGGAGTGTGCCTCATTTCGTGGGGCCTTT‑GGGACTTAGCCAGAATAATCAAGTCGTTACACGACACTAAGTGATAAACTCAAGGTCCCTAAATTAATACGACTCACtatctgtctcttatacacatctccga                                                                                                                                           >  1:106284/1‑278 (MQ=255)`  
                                                                                                                                                                                               `gCTGGTATGACCGAAGAGGATATTATCAAGCAGGGACAAATGGCTCGAATCCTACGGTTCAACGAGTACAACTTTATTGACAAG‑‑GAGATTTACCTGTGGAGACCGTAACGTATATTGGTCTGGGTCTTTGTGTTATCGGAGTGTGCCTCATTTCGGGGGGCCTTTGGGGACTTAGCCAGAATAATCAAGTCGTTACACGACACTAAGTGATCAACTACAGATCCCTAAATAAATACGACTAACTATAGGGAGCTAGGGGCCTGTACGATTATTAATTTAAGATTTAACCCTAAGGAGAAtt                                                                                                           >  1:155214/1‑300 (MQ=255)`  
                                                                                                                                                                                                  `ggTATGACCGAAGAGGATATTATCAAGCAGGGCCAAATAGCTCGAATCCTACGGTTCAACGAGTACAACTTTATTGACAAG‑‑GAGATTTACCTGTGGAGACCGTAACGTATATTGGTCTGGGTCTTTGTGTTCTCGGAGTGTGCCTCATTTCGTGGGGCCTTT‑GGGACTTAGCCAGAATAATCAAGTCGTTACACGACACTAAGTGATAAACTCAAGGTCCCTAAATTAATACGACTCACTATAGGGAGATAGGGGCCTTTACGATTATTACTTTAAGATTTAACTCTAAGAGGAATCtttt                                                                                                       >  1:252412/1‑300 (MQ=255)`  
                                                                                                                                                                                                   `gTATGACCGAAGAGGATATTATCAAGCAGGGCCAAATGGCTCGAATCCTACGGTTCAACGAGTACAACTTTATTGACAAG‑‑GAGATTTACCTGTGGAGACCGTAACGTATATTGGTCTGGGTCTTTGTGTTCTCGGAGTGTGCCTCATTTCGTGGGGCCTTT‑GGGACTTAGCCAGAATAATCAAGTCGTTACACGACACTAAGTGATAAACTCAAGGTCCCTAAATTAATACGACTCACTATAGGGAGATAGGGGCCTTTACGATTATTACTTTAAGATTTAACTCTAAGAGGAATCTttat                                                                                                      <  1:74200/301‑1 (MQ=255)`  
                                                                                                                                                                                                   `gTATGACCGAAGAGGATATTATCAAGCAGGACCAAATGGCTCGAATCCTACGGTTCAACGAGTACAACTTTATTGACAAG‑‑GAGATTTACCTGTGGAGACCGTAACGTATATTGGTCTGGGTCTTTGTGTTCTCGGAGTGTGCCTCATTTCGTGGGGCCTTT‑GGGACTTAGCCAGAATAATCAAGTCGTTACACGACACTAAGTGATAAACTCAAGGTCCCTAAATTAATACGACTCACTATAGGGAGATAGGGGCCTTTACGATTATTACTTTAAGATTTAACTCTAAGAGGAATCTttat                                                                                                      <  1:344741/301‑1 (MQ=255)`  
                                                                                                                                                                                                       `gACCGAAGAGGATATTATCAAGCAGGGCCAAATGGCTCGAATCCTACGGTTCAACGAGTACAACTTTATTGACAAG‑‑GAGATTTAACTGTGGAGACCGTAACGTATATTGGTCTGGGTCTTTGTGTTCTCGGAGTGTGCCTCATTTCGTGGGGCCTTT‑GGGACTTAGCCAGAATAATCAAGTCGTTACACGACACTAAGTGATAAACTCAAGGTCCCTAAATTAATACGACTCACTATAGGGAGATAGGGGCCTTTACGATTATTACTTTAAGATTTAACTCTAAGAGGAATCTTTATTATg                                                                                                  <  1:70974/301‑1 (MQ=255)`  
                                                                                                                                                                                                        `atcGAAAATGATATGAACAAGCAGGGCCAAATGGCTCGAATCCTACTGTTCAACGAGTACAACTTTATTGACAAG‑‑GAGATTTACCTGTGGATGCCGTAACGTATATTGGTCTGGGTCTTTGTGTTCTTGGAGTGGGCCTCATTTCGTGGGGCCTTT‑GGGACTTTGCCAGAATGATCAAGTCGTTACACGACACTAAGTGATAAACTCAAGGTCCCTAAATTAATACGACTAACTATAGGGGGATAGGTGCCTTTACGATTATTACTTTAAGATTTAACTCTAAGAGGAATCTTTATTATGt                                                                                                 <  1:150162/299‑1 (MQ=255)`  
                                                                                                                                                                                                             `agagGATATTATCAAGCAGGGCCAAATGACTCGAATCCTACGGTTCAACGAGTACAACTTTACTGACAAG‑‑GAGATTTACCTGTGGAGACCGTAACGTATATTGGTCTGGGTCTTTGTGTTCTCGGAGTGTGCCTCATTTCGTGGGGCCTTT‑GGGACTTAGCCAGAATAATCAAGTCGTTACACGACACTAAGTGATAAACTCAAGGTCCCTAAATTAATACGACTCACTATAGGGAGATAGGGGCCTTTACGATTATTACTTTAAGATTTAACTCTAAGAGGAATCTTTATTATGTTAaca                                                                                            <  1:57440/301‑1 (MQ=255)`  
                                                                                                                                                                                                                `ggATATTATCAAGCAGGGCCAAATGGCTCGAATCCTACGGTTCAACGAGTACAACTTTATTGACAAG‑‑GAGATTTACCTGTGGAGACCGTAACGTATATTGGTCTGGGTCTTTGTGTTCTCGGAGTGTGCCTCATTTCGTGGGGCCTTT‑GGGACTTAGCCAGAATAATCAAGTCGTTACACGACACTAAGTGATAAACTCAAGGTCCCTAAATTAATACGACTCACTATAGGGGTATAGGGGCCTTTACGATTATTACTTTAAGATTTAACTCTAAGAGGAATCTTTATTATGTTAACACCt                                                                                         >  1:302760/1‑301 (MQ=255)`  
                                                                                                                                                                                                                `ggATATTATCAAGCAGGGCCAAATGGCTCGAATCCTACGGTTCAACGAGTACAACTTTATTGACAAG‑‑GAGATTTACCTGTGGAGACCGTAACGTATATTGGTCTGGGTCTTTGTGTTCTCGGAGTGTGCCTCATTTCGTGGGGCCTTT‑GGGACTTAGCCAGAATAATCAAGTCGTTACACGACACTAAGTGATAAACTCAAGGTCCCTAAATTAATACGACTCACTATAGGGAGATAGGGGCCTTGACGATTATTACTTTAAGATTTAACTCTAAGAGGAATCTTTATTATGtctgtctct                                                                                         >  1:121461/1‑293 (MQ=255)`  
                                                                                                                                                                                                                `ggATATTATCAAGCAGGGCCAAATGGCTCGAATCCTACGGCTCAACGAGTACAACTTTATTGACAAG‑‑GAGATTTACCTGAGGAGACCGTAACGTATATTGGTCTGGGTCTTTGTGTTCTCGGAGTGTGCCTCATTTCGTGGGGCCGTT‑GTGACTTAGCCAGAATAATCAAGTCGTTACACGGCACTAAGTGATGAACTTAAGGTCCCTAAATTAATACGACTCACTATAGGGAGCTAGGGGCCTTTACGATTATTACTTTAAGAATTAACTCTAAGAGGAATCTTTATTATGTTAACACCt                                                                                         >  1:18401/1‑301 (MQ=255)`  
                                                                                                                                                                                                                   `aattatCAAGCAGGGCCAAATGGCTCGAATCCTACGGTTCAACGAGTACAACTTTATTGACAAG‑‑GAGATTTACCTGTGGAGACCGTAACGTATATTGGTCTGGGTCTTTGTGTTCTCGGAGTGTGCCTCATTTCGTGGGGCCTTT‑GGGACTTAGCCAGAATAATCAAGTCGTTACACGACACTAAGTGAAAAACTCAAGGTCCCTAAATTAATACGACTCACTATAGGGAGATAGGGGCCTTTACGATTATTACTTTAAGATTTAACTCTAAGAGGAATCTTTATTATGTTAACACCTAtt                                                                                      <  1:224957/300‑1 (MQ=255)`  
                                                                                                                                                                                                                     `ttatCAAGCAGGGCCAAATGGCTCGAATCCTACGGTTCAACGAGTACAACTTTATTGACAAG‑‑GAGATTTACCTGTGGAGACCGTAACGTATATTGGTCTGGGTCTTTGTGTTCTCGGAGTGTGCCTCATTTCGTGGGGCCTTT‑GGGACTTAGCCAGAATAATCAAGTCGTTACACGACACTAAGTGATAAACTCACGGTCCCTAAATTAATACGACTCACTATAGGGAGATAGGGGCCTTTACGATTATTACTTTAAGATTTAACTCTAAAAGGAATCTTTATTATGTTAACACCTATTaa                                                                                    >  1:110011/1‑301 (MQ=255)`  
                                                                                                                                                                                                                     `ttatCAAGCAGGGCCAAATGGCTCGAATCCTACGGTTCAACGAGTACAACTTTATTGACAAG‑‑GAGATTTACCTGTGGAGACCGTAACGTATATTGGTCTGGGTCTTTGTGTTCTCGGAGTGTGCCTCATTTCGTGGGGCCTTT‑GGGACTTAGCCAGAATAATCAAGTCGTTACACGACACTAAGTGATAAACTCAAGGTCCCTAAATTAATACGACTCACTATAGGGAGATAGGGGCCTTTACGATTATTACTTTAAGATTTAACTCTAAGAGGAATCTTTATTATGTTAACACCTATTaa                                                                                    >  1:96434/1‑301 (MQ=255)`  
                                                                                                                                                                                                                     `ttatCAAGCAGGGCCAAATGGCTCGAATCCTACGGTTCAACGAGTACAACTTTATTGACAAG‑‑GAGATTTACCTGTGGAGACCGTAACGTATATTGGTCTGGGTCTTTGTGTTCTCGGAGTGTGCCTCATTTCGTGGGGCCTTT‑GGGACTTAGCCAGAATAATCAAGTCGTTACACGACACTAAGTGATAAACTCAAGGTCCCTAAATTAATACGACTCACTATAGGGAGATAGGGGCCTTTACGATTATTACTTTAAGATTTAACTCTAAGAGGAATCTTTATTATGTTAACACCTATTaa                                                                                    >  1:270907/1‑301 (MQ=255)`  
                                                                                                                                                                                                                      `tatCAAGCAGGGCCAAATGGCTCGAATCCTACGGTTCAACGAGTACAACTTTATTGACAAG‑‑GAGATTTACCTGTGGAGACCGTAACGTATATTGGTCTGGGTCTTTGTGTTCTCGGAGTGTGCCTCATTTCGTGGGGCCTTT‑GGGACTTAGCCAGAATAATCAAGTCGTTACACGACACTAAGTGATAAACTCAAGGTCCCTAAATTAATACGACTCACTATAGGGAGATAGGGGCCTTTACGATTATTACTTTAAGATTTAACTCTAAGAGGAATCTTTATTATGTTAACACCTATTAAc                                                                                   <  1:283300/301‑1 (MQ=255)`  
                                                                                                                                                                                                                      `tatCAAGCAGGGCCAAATGGCTCGAATCCTACGGTTCAACGAGTACAACTTTATTGACAAG‑‑GAGATTTACCTGTGGAGACCGTAACGTATATTGGTCTGGGTCTTTGTGTTCTCGGAGTGTGCCTCATTTCGT‑GGGCCTTT‑GGGACTTAGCCAGAATAATCAAGTCGTTACACGACACTAAGTGATAAACTCAAGGTCCCTAAATTAATACGACTCACTATAGGGAGATAGGGGCCTTTACGATTATTACTTTAAGATTTAACTCTAAGAGGAATCTTTATTATGTTAACACCTATTAAcc                                                                                  <  1:330663/301‑1 (MQ=255)`  
                                                                                                                                                                                                                      `tatCAAGCAGGGCCAAATGGCTCGAATCCTACGGATCAACGAGTACAACTTTAGTGACAAG‑‑GAGATTTACCTGTGGAGACCGTAACGTATATTGGTCTGGGTCTTTGTGTTCTCGGAGTGTGCCTCATTTCGTGGGGCCTTT‑GGGACTTAGCCAGAATAATCAAGTCGTTACACGACACTAAGTGATAAACTCAAGGTCCCTAAATTAATACGACTCACTATAGGTAGATAGGGGCCTTTACGATTATTACTTTAAGATTTAACTCTAAGAGGAATCTTTATTATGTTAACACCTATTAAc                                                                                   <  1:254298/301‑1 (MQ=255)`  
                                                                                                                                                                                                                      `tatCAAGCAGGGCCAAAGGGCTCGAATCCTACGGTTCAACGAGTACAACTTTATTGACAAG‑‑GAGATTTACCTGTGGAGACCGTAACGTATATTGGTCTGGGTCTTTGTGTTCTCGGAGTGTGCCTCATTTCGTGGGGCCTTT‑GGGACTTAGCCAGAATAATCAAGTCGTTACACGACACTAAGTGATAAACTCAAGGTCCCTAAATTAATACGACTCACTATAGGGAGATAGGGGCCTTTACGATTATTACTTTAAGATTTAACTCTAAGAGGAATCTTTATTATGTTAACACCTATTAAc                                                                                   <  1:20102/301‑1 (MQ=255)`  
                                                                                                                                                                                                                      `aagagacaggggCCAAATGGCTCGAATCCTACGGTTCAACGAGTACAACTTTATTGACAAG‑‑GAGATTTACCTGTGGAGACCGTAACGTATATTGGTCTGGGTCTTTGTGTTCTCGGAGTGTGCCTCATTTCGTTGGGCCTTT‑GGGACTTAGCCAGAATAATCAAGTCGTTACACGACACTAAGTGATAAACTCAAGGTCCCTAAATTAATACGACTCACTATAGGGAGATAGGGGCCTTTACGATTATTACTTTAAGATTTAACTCTAAGAGGAATCTTTATTATGTTAACACCTATTAAc                                                                                   <  1:334626/292‑1 (MQ=255)`  
                                                                                                                                                                                                                      `aaacAAGCAGGGACAAATGGCTCGAATCCTACGGTTCAACGAGTACAACTTTATTGACAAG‑‑GAGATTTACCTGTGGAGACCGTAACGTATATTGGTCTGGGTCTTTGTGTTCTCGGAGTGTGCCTCATTTCGTGGGGCCTTT‑GGGACTTAGCCAGAATAATCAAGTCGTTACACGACACTAAGTGATAAACTCAAGGCCCCTAAATTAATACGACTCACTATAGGGAGATAGGGGCCTTTACGATTATTACTTTAAGATTTAACTCTAAGAGGAATCTTTATTATGTTAACACCTATTAAc                                                                                   <  1:188695/298‑1 (MQ=255)`  
                                                                                                                                                                                                                       `atCAAGTAGGGCCAAATGGCTCGAATCCTACGGTTCAACGAGTACAACTTTATTGACAAG‑‑GAGATTTACCTGTGGAGACCGTAACGTATATTGGTCTGGGTCTTTGTGTTCTCGGAGTGTGCCTCATTTCGTGGGGCCTTT‑GGGACTTAGCCAGAATAATCAAGTCGTTACACGACACTAAGTGATAAACTCAAGGTCCCTAAATTAATACGACTCACTATAGGGAGATAGGGGCCTTTACGATTATTACTTTAAGATTTAACTCTAAGAGGAATCTTTATTATGTTAACACCTATTAAcc                                                                                  <  1:125929/301‑1 (MQ=255)`  
                                                                                                                                                                                                                             `cAGGGCCAAATGGCTCGAATCCTACGGTTCAACGAGTACAACTTTATTGACAAG‑‑GAGATTTACCTGTGGAGACCGTAACGTATATTGGTCTGGGTCTTTGTGTTCTCGGAGTGTGCCTCATTTCGTGGGGCCTTT‑GGGACTTAGCCAGAATAATCAAGTCGTTACACGACACTAAGTGATAAACTCAAGGTCCCTAAATTAATACGACTCACTATAGGGAGATAGGGGCCTTTACGATTATTACTTTAAGATTTAACTCTAAGAGGAATCTTTATTATGTTAACACCTATTAACCAATTAc                                                                            <  1:5423/301‑1 (MQ=255)`  
                                                                                                                                                                                                                                `ggCCAAATGGCTCGAATCCTACGGTTCAACGAGTACAACTTTATTGACAAG‑‑GAGATTTACCTGTGGTGACCGTAACGTATATTGGTCTGGGTCTTTGTGTTCTCGGAGTGTGCCTCATTTCGTGGGGCCTTT‑GGGACTTAGCCAGAATAATCAAGTCGTTACACGACACTAAGTGATAAACTCAAGGTCCCTAAATTAATACGACTCACTATAGGGAGATAGGGGCCTTTACGATTATTACTTTAAGATTTAACTCTAAGAGGAATCTttatttgtctcttatacacatctcctagcccac                                                                         >  1:328211/1‑273 (MQ=255)`  
                                                                                                                                                                                                                                      `atgtgtataagagacaggTTCAACGAGTACAACTTTATTGACAAG‑‑GAGAGTTACCTGTGGAGACAGGAACGTATATTGGTCTGGGTCTTTGTGTTCTCGGAGTGGGCCTCATTTCGTGGGGCATTT‑GGGACTTAGCCAGAATAATCAAGTAGTTACACGACACTAAGTGATAAACTCAAGGTCCCTAAATTAATACGACTCAATATAGGGAGATAGGGGCCTTTACGATTATTACTTTAAGATTTAACTCTAAGAGGAATCTTTATTATGTTAACACCTATTAACCAATTACTTAAGAAcc                                                                   <  1:134518/285‑1 (MQ=255)`  
                                                                                                                                                                                                                                      `aTGGATAGAATCCTACGGTTCAACGAGTAAAACTATATAGAAAAG‑‑GAGATTAACCTGTGGAGACCGTAACGTATATTGGTCGGGGTCTTTGTGTTCACGGAGTGAGCCAAATTTCGTGGGGCCTTT‑GGGACTTAGCCAGAATAATCAAGTCGTTACACGAAACTAAGTGATAAACTAAAGGTCCCTAAATTAATACGACTCACTATAGGGAGATAGGGGCCTTTACGATTATTACTTTAAGATTTAACTCTAAGAGGAATCTTTATTATGTTAACACCTATTAACCAATTACTTAAGAAcc                                                                   <  1:233686/301‑1 (MQ=255)`  
                                                                                                                                                                                                                                                `tCCTACGGTTCAACGAGTACAACTTTATTGACAAG‑‑GAGATTTACCTGTGGAGACCGTAACGTATATTGGTCTGGGTCTTTGTGTTCTCGGAGTGTGCCTCATTTCGTGGGGCCTTT‑GGGACTTAGCCAGAATAATCAAGTCGTTACACGACACTAAGTGATAAACTCAAGGTCCCTAAATTAATACGACTCACTATAGGGAGATAGGGGCCTTTACGATTATTACTTTAAGATTTAACTCTAAGAGGAATCTTTATTATGTTAACACCTATTAACCAATTACTTAAGAACCCTAACGatat                                                         >  1:103388/1‑301 (MQ=255)`  
                                                                                                                                                                                                                                                   `tACGGTTCAACGAGTACAACTTTATTGACAAG‑‑GAGATTTACCTGGGGAGACCGTAACGTATATTGGTCTGGGTCTTTGTGTTCTCGGAGTTTGCCTCATTTCGTGGGGCCTTT‑GGGACTTCGCCAGAATAATCAAGTCGTTACACGACACTAAGTGATAAACTCAAGGTCCCTAAATTAATACGACTCACTATAGGGAGATAGGGGCCTTTACGATTATTACTTTAAGATTTAACTCTAAGAGGAATCTTTATTATGTTAACACCTATTAACCAATTACTTAAGAACCCTAACGATATTcc                                                      <  1:273639/301‑1 (MQ=255)`  
                                                                                                                                                                                                                                                     `gggTTCAACGAGTACAACTTTATTTACAAG‑‑GAGATTTACCTTTGGAGACCGTAACGTATATTGGTCTGGGTCTTTGTGTTCTCGGAGTGTGCCTCATTTCGTGGGGCCTTT‑GGGACTTAGCCAGAATAATCAAGTCGTTACACGACACTAAGTGATAAACTCAAGGTCCCTAAATTAATACGACTCACTATAGGGAGATAGGGGCCTTTACGATTATTACTTTAAGATTTAACTCTAAGAGGAATCTTTATTATGTTAACACCTATTAACCAATTACTTAAGAACCCTAACGATATTCCAg                                                    <  1:346695/300‑1 (MQ=255)`  
                                                                                                                                                                                                                                                             `ggAGTACAAAGTTTGTGACAAG‑‑GAGATTTACCTGTTGAGACCGTAACGTATATTGGTCTGGGACTTTGTGTTCTCGTAGTGTGCCTCATTTCGTGGGGCCTTT‑GGGACTTAGCCAGAATAATCAAGTCGTTACACGACACTAAGTGATAAACTCAAGGTCCCTAAATTAATACGACTCACTATAGGGAGATAGGGGCCTTTACGATTATTACTTTAAGATTTAACTCTAAGAGGAATCTTTATTATTTTAACACCTATTAACCAATTACTTAAGAACCCTAACGATATTCCAGATGTACCt                                            <  1:193138/300‑1 (MQ=255)`  
                                                                                                                                                                                                                                                             `agAGTACAACTTTATTGAAAAG‑‑GAGATTTACCTGTGGAGACCGTAACGTATAGTGGTCTGGGTCTTTGTGTTTTCGGAGTGTGCCTCATGTCGTGGGGCCTTT‑GGGACTTAGCCAGAATAATCAAGTCGTTACACGACACTAAGTGATAAACTCAAGGTCCCTAAATTAATACGACTCACTATAGGGAGATAGGGGCCTTTACGATTATTACTTTAAGATTTAACTCTAAGAGGAATCTTTATTATGTTAACACCTATTAACCAATTACTTAAGAACCCTAACGATATTCCAGATGTACCt                                            <  1:162407/300‑1 (MQ=255)`  
                                                                                                                                                                                                                                                                                `ataagagacagtACCTGTGGAGACCGTAACGTATATTGGTCTGGGTCTTTGTGTTCTCGGAGTGTGCCTCATTTCGTGGGGCCTTT‑GGGACTTAGCCAGAATAATCAAGTCGTTACACGACACTAAGTGATAAACTCAAGGTCCCTAAATTAATACGACTCACTATAGGGAGATAGGGGCCTTTACGATTATTACTTTAAGATTTAACTCTAAGAGGAATCTTTATTATGTTAACACCTATTAACCAATTACTTAAGAACCCTAACGATATTCCAGATGTACCTCGTGCAACCGCTGAGTa                           <  1:8018/290‑1 (MQ=255)`  
                                                                                                                                                                                                                                                                                `aaG‑‑GAGATTTACCTGTGGAGACCGTAACGTATATTGGTCTGGGTCTTTGTGTTCTCGGAGTGTGCCTCATTTCGTGGGGCCTTT‑GGGACTTAGCCAGAATAATCAAGTCGTTACACGACACTAAGTGATAAACTCAAGGTCCCTAAATTAATACGACTCACTATAGGGAGATAGGGGCCTTTACGATTATTACTTTAAGATTTAACTCTAAGAGGAATCTTTATTATGTTAACACCTATTAACCAATTACTTAAGAACCCTAACGATATTCCAGATGTACCTCGTGCAACCGCTGAGTATc                         <  1:262853/301‑1 (MQ=255)`  
                                                                                                                                                                                                                                                                                     `gagaTTTACCTGTGGAGACCGTAACGTTTATTGGTCTGGGTCTTTGTGTTCTCGGAGTGTGCCTCATTTCGTGGGGCCTTT‑GGGACTTAGCCAGAATAATCAAGTCGTTACACGACACTAAGTGAGAAACTCAAGGTCCCTAAATTAATACGACTCACTATAGGGAGATAGGGGCCTTTACGATTATTACTTTAAGATTTAACTCTAAGAGGAATCTTTATTATGTTAACACCTATTAACCAATTACTTAAGAACCCTAACGATATTCCAGATGTACCTCGTGCAACCGCTGAGTATCTAc                      <  1:6703/301‑1 (MQ=255)`  
                                                                                                                                                                                                                                                                                     `gagaTTTACCTGTGGAGACCGTAACGTATATTGGTCTGGGTCTTTGTGTTCTCGGAGTGTGCCTCATTTCGTGGGGCCTTT‑GGGACTTAGCCAGAATAATCAAGTCGTTACACGACACTAAGTGATAAACTCAAGGTCCCTAAATTAATACGACTCACTATAGGGAGATGGGGGCCTTTACGATTATTACTTTAAGATTTAACTCTAAGAGGAATCTTTATTATGTTAACACCTATTAACCAATTACTTAAGAACCCTAACGATATTCCAGATGTACCTCGTGCAAACGCTGAGtctgtct                      >  1:82234/1‑295 (MQ=255)`  
                                                                                                                                                                                                                                                                                     `gagaTTTACCTGTGGAGACCGTAACGTATATTGGTCTGGGTCTTTGTGTTCTCGGAGTGTGCCTCATTTCGTGGGGCCTTT‑GGGACTTAGCCAGAATAATCAAGTCGTTACACGACACTAAGTGATAAACTCAAGGTCCCTAAATTAATACGACTCACTATAGGGAGATAGGGGCCTTTACGATTATTACTTTAAGATTTAACTCTAAGAGGAATCTTTATTATGTTAACACCTATTAACCAATTACTTAAGAACCCTAACGATATTCCAGATGTACCTCGTGCAACCACTGAGTATCTAc                      <  1:39553/301‑1 (MQ=255)`  
                                                                                                                                                                                                                                                                                       `gaTTTACCTGTGGAGACCGTAACGTATATTGGTCTGGGTCTTTGTGTTCTCGGAGTGTGCCTCATTTCGTGGGGCCTTT‑GGGACTTAGCCAGAATAATCAAGTCGTTACACGACACTAAGTGATAATCTCAAGGTCCCTAAATTAATACGACTCACTCTAGGGAGATAGGGGCCTTTACGATTATTACTTTAAGATTTAACTCTAAGAGGAATCTTTATTATGTTAACACCTATTAACCAATTACTTAATAACCCTAACGATATTCCAGATGTACCTCTTGCAACCGCTGCGTATCTACAg                    >  1:341249/1‑301 (MQ=255)`  
                                                                                                                                                                                                                                                                                       `gaTTGACCTGTGGAGACCGGAACGTAAATTGGTCTGGGTCTTTGTGTTCTCGGAGTGTGCCTCATGTCGTGGGGCCTTT‑GGGACTTAGCCAGGATAATCAAGTCGTTACAAGAAACTAAGTGATAAACTCAAGGTCCCTAAATTAATACGACTCACTATAGGGAGATAGGGGCCTTTACGATTATTACTTTAAGATTTAACTCTAAGAGGAATCTTTATTATGTGAACACCTATTAACCAATTACTGAAGAACCCTAACGATATTCCAGATGTACCTCGTGCAACCGCTGAGTATCTACAg                    <  1:240302/301‑1 (MQ=255)`  
                                                                                                                                                                                                                                                                                        `aTTTACCTGTGGAGACCGTAACGTATATTGGTCTGGGTCTTTGTGTTCTCGGAGTGTGCCTCATTTCGTGGGGCCTTT‑GGGACTTAGCCAGAATAATCAAGTCGTTACACGACACTAAGTGATAAACTCAAGGTCCCTAAATTAATACGACTCACTATAGGGAGATAGGGGCCTTTACGATTATTACTTTAAGATTTAACTCTAAGAGGAATCTTTATTATGTTAACACCTATTAACCAATTACTTAAGAACCCTAACGATATTCCAGATGTACCTCGTGCAACCGCTGAGTATCTACAgg                   <  1:295839/301‑1 (MQ=255)`  
                                                                                                                                                                                                                                                                                        `aTTTACCTGTGGAGACCGTAACGTATATTGGTCTGGGTCTTTGTGTTCTCGGAGTGTGCCTCATTTCGTGGGGCCTTT‑GGGACTTAGCCAGAATAATCAAGTCGTTACACGACACTAAGTGATAAACTCAAGGTCCCTAAATTAATACGACTCACTATAGGGAGATAGGGGCCTTTACGATTATTACTTTAAGATTTAACTCTAAGAGGAATCTTTATTATGTTAACACCTATTAACCAATTACTTAAGAACCCTAACGATATTCCAGATGTACCTCGTGCAACCGCTGAGTATCTACAgg                   <  1:276125/301‑1 (MQ=255)`  
                                                                                                                                                                                                                                                                                           `tACCTGTGGAGACCGTAACGTATATTGGTCTGGGTCTTTGTGTTCTCGGAGTGTGCCTCATTTCGTGGGGCCTTT‑GGGACTTAGCCAGAATAATCAAGTCGTTACACGACACTAAGTGATAAACTCAAGGTCCCTAAATTAATACGACTCACTATAGGGAGATAGGGGCCTTTACGATTATTACTTTAAGATTTAACTCTAAGAGGAATCTTTATTATGTTAACACCTATTAACCAATTACTTAAGAACCCTAACGATATTCCAGATGTACCTCGTGCAACCGCTgtgtctgtctcttata                >  1:108716/1‑289 (MQ=255)`  
                                                                                                                                                                                                                                                                                                      `aCCGTAACGTATATTGGTCTGGGTCTTTGTGTTCTCGGAGTGTGCCTCATTTCGTGGGGCCTTT‑GGGACTTAGCCAGAATAATCAAGTCGTTACACGACACTAAGTGATAAACTCAAGGTCCCTAAATTAATACGACTCACTATAGGGAGATAGGGGCCTTTACGATTATTACTTTAAGATTTAACTCTAAGAGGAATCTTTATTATGTTAACACCTATTAACCAATTACTTAAGAACCCTAACGATATTCCAGATGTACCTCGTGCAACCGCTGAGTATCTACAGGTTCGATTCAACTAt     >  1:77034/1‑301 (MQ=255)`  
                                                                                                                                                                                                                                                                                                       `ccGTAACGTATATTGGTCTGGGTCTTTGTGTTCTCGGAGTGTGCCTCATTTCGTGGGGCCTTT‑GGGACTTAGCCAGAATAATCAAGTCGTTACACGACACTAAGTGATAAACTCAAGGTCCCTAAATTAATACGACTCACTATAGGGAGATAGGGGCCTTTACGATTATTACTTTAATATTTAACTCTAAGAGGAATCTTTATTATGTTAACACCTATTAACCAATTACTTAAGAACCCTAACGATATTCCAGATGTACCTCGTGCAACCGCTGAGTATCTACAGGTTCGATTCAACTATg    >  1:88749/1‑301 (MQ=255)`  

                                                                                                                                                                                                                                                                                                            `|`                                                                                                                                                                                                                                                                                                            
`ATGGTTACTCAGGGATTGCTGGATGGGGTGATACCGCCGAGGACTTCTTGAATAACCCGTTCATAACCGAGCCTAAAACGTCTGTGCTTAAGTCCGGTAAGAACAAAGGCCAAGAGGTTACTAAATGGGTTAAACGCGACCCTGAGCCTCATGAGACGCTTTGGGACTGCATTAAGTCCATTGGCGCGAAGGCTGGTATGACCGAAGAGGATATTATCAAGCAGGGCCAAATGGCTCGAATCCTACGGTTCAACGAGTACAACTTTATTGACAAG‑‑GAGATTTACCTGTGGAGACCGTAGCGTATATTGGTCTGGGTCTTTGTGTTCTCGGAGTGTGCCTCATTTCGTGGGGCCTTT‑GGGACTTAGCCAGAATAATCAAGTCGTTACACGACACTAAGTGATAAACTCAAGGTCCCTAAATTAATACGACTCACTATAGGGAGATAGGGGCCTTTACGATTATTACTTTAAGATTTAACTCTAAGAGGAATCTTTATTATGTTAACACCTATTAACCAATTACTTAAGAACCCTAACGATATTCCAGATGTACCTCGTGCAACCGCTGAGTATCTACAGGTTCGATTCAACTATGCG  >  NC_001604.1/18108‑18703`

  

Alignment Legend

Aligned base mismatch/match (shaded by quality score): `ATCG/ATCG` `< 3 ≤` `ATCG/ATCG` `< 10 ≤` `ATCG/ATCG` `< 23 ≤` `ATCG/ATCG` `< 35 ≤` `ATCG/ATCG` `< 38 ≤` `ATCG/ATCG`

Unaligned base: `atcg`    Masked matching base: `atcg`    Alignment gap: `‑`    Deleted base: `‑`
